# Regulatory requirements for designing PET-CT facility in India

**DOI:** 10.4103/0972-3919.72684

**Published:** 2010

**Authors:** Pankaj Tandon

**Affiliations:** Radiological Safety Division, Atomic Energy Regulatory Board, Niyamak Bhavan, Anushakti Nagar, Mumbai - 400 094, India. E-mail: pantan@gmail.com

## INTRODUCTION

In India, cyclotron-produced radionuclides are gaining importance in molecular imaging in Nuclear Medicine (NM) departments. The importance of this modality among others is due to the fact that it provides valuable clinical information, which was lacking in other available modalities. Presently, every well-established hospital would like to procure Medical Cyclotron or positron emission tomography-computed tomography (PET-CT) facility in their NM department. Because cyclotron-produced radionuclides have higher energy than the other routinely used radionuclides for diagnosis, it becomes essential for the user to know about the regulatory requirement and radiation safety precautions that one has to take for the installation of this new modality in their premises. The various stages of approval of PET-CT facility by the Atomic Energy Regulatory Board (AERB) and important steps that one has to know/follow before planning for this new facility are summarized in the following sections.

## SITE AND LAYOUT PLAN APPROVAL

**Figure 1:**
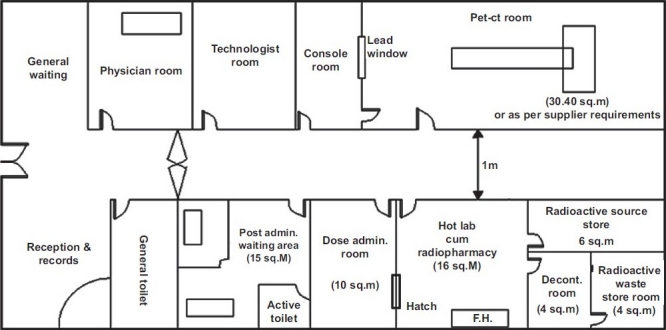
A typical lay-out of a positron emission tomography-computed tomography (PET-CT) facility. Note: All the walls of the PET-CT facility should be made of thick brick or thick concrete but the walls of PET-CT room should be concrete only, the thickness of which depends on the area and workload. (FH = fume hood; fume hood should be installed, if required)

**Figure 2:**
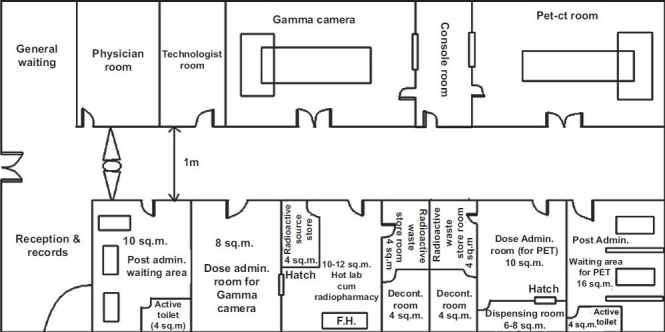
A typicallay out for nuclear medicine facility having both gamma camera and positron emission tomography-computed tomography (PET-CT) installations. Note: All the walls of the PET-CT facility should be made of thick brick or thick concrete but the walls of PET-CT room should be concrete only, the thickness of which depends on the area and workload. (FH, fume hood; fume hood should be installed, if required)

The user has to submit to AERB two copies each of the proposed layout plan, site plan, and elevation drawing of the facility indicating the floor, nature of occupancy around, above and below, if any, has to be submitted in “B3” size paper (353×500 mm^2^) along with the application form AERB/RSD/NMF/SLA (downloadable from www.aerb.gov.in). The user has to clearly indicate the dimension of each of the rooms associated with the facility in the proposed layout plan of the NM department. When the user has to plan the laboratory, it is required that the arrangement of the various rooms associated with the facility has to follow the principle of low active area to high active area, that is, entrance of the facility should have reception/general waiting area, and at the end hot laboratory cum radiopharmacy/radioactive waste storage area is to be planned. The typical layout plans for the facility given in Figures 1 and 2 may be referred to design the PET-CT facility alone or PET-CT facility along with the gamma camera facility with respect to the arrangement/allocation of rooms and area requirement. The above documents have to be submitted to the Head, Radiological Safety Division (RSD), AERB. On scrutinizing the plans from radiologic safety point of view, necessary approval of facility will be granted.

## SUBMISSION OF REGULATORY CONSENT FORM

The user has to submit the details of the completion of the construction work as per the approved plan, installation of equipments, procurement of radiologic protection accessories, enrollment of radiation workers in Personal Monitoring Services and availability of qualified staff as per AERB Safety Code AERB/SC/MED-IV (Rev-1 2001), shall be intimated to Head, RSD, AERB, by submitting the Regulatory Consent form no. AERB/444-NM/RC-FORM (downloadable from www.aerb.gov.in). The Radiological Safety Officer (RSO) as per the qualification mentioned in the AERB Safety Code AERB/SC/MED-IV (Rev-1 2001) has to be nominated by the employer for the NM facility by submitting the application form no. AERB/441/RSOM-II/III (downloadable from www.aerb.gov.in).

## PRECOMMISSIONING INSPECTION

In this stage, the precommissioning inspection of the facility will be carried out by AERB official(s) to ensure that the construction of NM facility is as per the approved plan and also to verify the information provided in stage “2” mentioned above.

## APPROVAL FOR COMMISSIONING/ROUTINE OPERATION

On ensuring the compliance of the requirement as specified in AERB safety Code SC/MED-IV (Rev.1. 2001) for the safe handling of radioactive material in the approved NM facility, the authorization for the procurement of radioactive material indigenously or no objection certificate (NOC) for procurement of radioactive material from abroad will be issued for the stipulated time period. The renewal for the same is done only after receipt and review of the Annual Status Report AERB/NM/Radiation Safety/02 (downloadable from www.aerb.gov.in) by AERB.

## WORK PRACTICE

In NM facility, the radiopharmaceutic formulation should be prepared, handled, administered to the patients, and disposed of in a safe manner taking into account adequate radiation protection measures. Radioisotopes should be stored, used, and transported safely and securely all the time. Any unusual event that has resulted or has the potential to result in overexposure to the workers or public should be reported to the AERB. Annual Safety Status report of the facility should reach the AERB in the prescribed format at the end of each calendar year. Any change in the qualified person or design of the facility shall be reported to the AERB. Cooperation should be extended to the authorized inspectors from the AERB during inspection of the facility. Failure of compliance to radiation safety procedures may attract enforcement action by the AERB.

## WASTE DISPOSAL

In India, radioactive waste management is governed by the Atomic Energy (Safe Disposal of Radioactive Wastes) Rules, 1987, GSR-125 issued under the Atomic Energy Act, 1962. Chairman, AERB is the competent authority. Implementation of the Rules is primarily to ensure safety of the public and the environment.

Radioactive waste needs to be managed safely because it is potentially hazardous to human health and the environment. Through good practices in the production and use of radionuclides, the amount of waste may be significantly reduced but not fully eliminated. It is important that safe waste management, in compliance with all relevant regulations is considered and planned for at the early stages of any projects involving radioactive materials. The radioactive waste in hospitals comprises different types of waste. It may be in solid, liquid, or gaseous form. The radioactive material may be mixed with different chemicals, which in itself would be hazardous or flammable. Moreover, some of the waste is mixed with biological substances, such as blood and may be infectious. Also, special precautions must be taken for disposing items, such as needles used for injection. All these aspects must be accounted for in the planning of waste treatment in a hospital. In NM, having PET-CT facility, mostly short-lived radioisotopes C-11, N-13, O-15, F-18, and others are used in unsealed form. The half-lives of these radioisotopes range from few minutes to few hours. In a typical NM department, there may be certain sealed sources used for calibration of dose calibrators, survey instruments; these sources may end up as spent sources after their useful life or during the decommissioning of the department. Gaseous radioactive waste arising from the PET-CT department may be very rare.

The solid waste generated in NM includes cover papers, gloves, contaminated items, empty vials, and syringes. The liquid waste may comprise unused or leftover radiopharmaceutic solutions, radioactive spills, decontamination effluents, laboratory washings, and so on.

All the labels on the containers having contaminated radioactive material should be removed defaced prior to disposal. There are 2 main approaches to the disposal of radioactive waste. One is characterized as “dilute and disperse” and the other as “confine and contain.” By the “dilute and disperse” concept, radioactive material, in aqueous or gaseous form, is released into the environment in such a way that the material is diluted and distributed over a large volume so that the final concentration of radionuclides is acceptably low. In the “confine and contain” approach, the waste is collected and converted into a form such that, when placed in a repository, it will retain the radionuclides until the activity has decayed, or at least will ensure that the leakage of radionuclides from the repository does not give rise to unacceptable concentrations anywhere in the environment. This approach is always used for longer-lived solid radioactive waste and spent sources, which are negligible in the PET-CT facility.

## DECOMMISSIONING

When the NM facility is no longer to be used, the permission for decommissioning should be obtained from the AERB.

## RADIATION PROTECTION ASPECTS OF PET-CT FACILITIES

NM facility with PET-CT employs relatively large activities of high-energy photon emitting radioisotopes. This coupled with the current dose limits for members of the public, can result in a shielding requirement. Even modest reductions in the radiation levels at 511 keV require significant amounts of shielding. A thorough and site-specific evaluation has to be made for each facility.

## SHIELDING CALCULATIONS

Presently, AAPM task group report no. 108 on PET and PET-CT shielding requirements are being used for carrying out the shielding calculations. In outline, the sequence of steps similar to any other shielding calculation is as follows:

- Obtain an architectural layout of the facility having dimensions of each of the rooms associated with the facility.
- Determine the expected workload (number of patients per week) of the facility, maximum radioactivity to be administered per procedure, and CT workload (total mAs and kVp) per patient.
- Determine the occupancies of areas within the facility and in adjacent, uncontrolled areas. Include consideration of occupancies at floors above and below the facility.
- Determine the location of the place where all the initial activities of radioisotopes are to be dispensed. This also includes the injected patient working area.
- Obtain the isodose curves of the transmission sources in a PET-CT scanner.
- Obtain the total dose from all the sources at test points established at the principal work areas and at points in uncontrolled area using the source strengths, source locations, workload factors, gamma-ray dose constants, and the inverse square law.
- If the shielding calculation does not meet the protection criteria, additional shielding materials are to be used.

## RADIATION SAFETY ISSUES

Vendors are providing specialized equipment to reduce exposure to operating personnel in the PET-CT facility and to improve instrument performance in the higher radiation background found in the hot laboratory. The equipment include:

- Automated dose dispenser to reduce the dose to the technologist while dispensing the unit dose for patient administration.
- Dose calibrator with thick lead shielding to reduce technologist exposure during the dose assay.
- Well counters with external shields to reduce background from the stored doses.
- Sources in the scanners and calibration sources.
- Tungsten syringe shields to reduce finger dose during injection.
- Remotely actuated syringes that keep the syringe totally enclosed in a shield while the operator delivers the dose by pushing on an extension rod.
- Extra thick L-block table-top shields (5 cm of lead compared with 1.2 cm of lead in standard NM applications), and
- Syringe carriers for transporting the dose from one room to another.

## OPERATING SUGGESTION

Transporting and positioning the patient are the operations that deliver significant exposure to the technologist. Maximize separation from the patient after injection and minimize the time spent with them. Patient instruction should be completed before injection, as should the completion of any forms or questionnaires. If the patient is ambulatory, allow as much separation as feasible when they are escorted to the scanner room. Minimize the time spent near the patient in the scanner room. Use of unit dose will reduce technologist exposure compared with the use of bulk distribution radiopharmaceutics. In the hot laboratory, particular attention should be paid to minimize the time required to handle the dose during the assay and verification steps.

It is always better to have the IV access with a butterfly infusion set before the dose is taken out of the shield to reduce the handling time of the syringe. Use of cart to transport the dose from the hot laboratory to the injection room will increase the separation between the technologist and the syringe and reduce the dose if the transport time is significant.

## PERSONNEL MONITORING SERVICES FOR STAFF MEMBERS

Every staff member, involved in radioactive work, such as radiopharmacy, radiochemistry, dispensing of radioactivity, radiopharmaceutic dose administration, patient imaging, and others, should be covered with personnel monitoring services. For availing this facility, one may approach the Head, CDandR Section, RP and AD, BARC, Anushakti Nagar, Mumbai – 400 094, or the respective accredited laboratory in their zone for the service. Presently, 3 such laboratories are accredited as given below.

1. M/s Avanttech Laboratories (P) Ltd.
  # 76, 7^th^ Street, Ground Floor,
  Porur Garden, Phase – I,
  Chennai – 600 095.
2. M/s Renentech Laboratories Pvt. Ltd.
  C-106, Synthofine Industrial Estate,
  Off Aarey Road, Goregaon (E)
  Mumbai – 40 063.
3. M/s Ultratech Laboratories Pvt. Ltd.
  12/15, Priyadarshini, Parisar (W)
  Bhilai, C.G. - 490020.

## RADIATION MONITORING DEVICES

In the NM department having the Medical Cyclotron and PET facilities, following are the monitoring equipments required:

1. GM-based survey meter/ionization chamber–based survey meter
2. Contamination monitor
3. Pocket dosimeters
4. Isotope dose calibrator.

## RADIATION SAFETY DEVICES

Since the quantity of radionuclide that is being handled is significant and the energy of associated radiation is high, it is absolutely necessary to have an automatic smaller dosage dispensing unit, fume hood, and L-Bench to handle smaller dose for quality assurance tests, lead bricks, shielding devices made of tungsten (syringe shield, source container, transport container, and others), remote handling devices (de-capper, cap sealer, long vial holder, pair of tongs, and others)

## STAFF REQUIREMENT FOR NUCLEAR MEDICINE LABORATORY

A facility to be approved by the AERB should have the following staff/personnel. The responsibilities that these personnel have to carry out are also given below.

### Nuclear medicine physician

#### Qualifications

- An MBBS degree recognized by the Medical Council of India; and
- A post graduate degree/diploma in NM recognized by the Medical Council of India or National Board of Examination, Ministry of Health and Family Welfare.

#### Responsibilities

The NM physician shall

- have the responsibility of dosage administration and maintenance of records providing name of the patient, nature of the procedure, radiopharmaceutic prescribed, quantity prescribed, name of the NM physician with signature and date, and name of the person administering the radiopharmaceutic with signature and date;
- prevent any possibility of misadministration and promptly report to the licensee and the competent authority in the event of any misadministration, adverse reaction or death of a patient administered with radioactivity;
- consider factors, such as proper choice of radiopharmaceutics, monitoring of procedure, and immobilization of the patient, to minimize radiation exposure to patient. Suitable methods for reducing absorbed dose to the patient shall be adopted;
- consider justification of diagnosis on pregnant patients/lactating mothers to limit the exposure to the fetus/infant not exceeding an absorbed dose of 1 mGy.
- adopt specific dosimetric consideration in pediatric patients to ascertain the risk–benefit ratio;
- inform patient on safety measures to be observed to avoid radiation exposure to the family members and others;
- ensure that where the quantity of radioactivity administered to a patient is in excess of the limits specified for radiopharmaceutics emitting gamma radiation (i) spread of contamination prevented and (ii) exposure of staff, other patients, and public minimized;

### Nuclear medicine technologist

#### Qualifications

- A Bachelor’s degree in NM technology from a university; or
- A Bachelor’s degree in Science from a university; and Bachelor degree/Post graduate degree/diploma in NM technology from a university.

#### Responsibilities

The NM technologist shall

- ensure proper functioning of all NM equipment, carry out periodic calibrations, quality assurance checks, and maintenance;
- ensure the radiopharmaceutic quality requirements, the route of administration, and the accuracy of dosage before giving it to a patient and take precautions to avoid misadministration;
- avoid spillage of radioactivity or contamination of the patient, premises, persons, and material by exercising care during dispensing/administration of radioactivity;
- report to RSO and the NM physician of any mishap in dispensing/administration of dosage to the patient or any unusual incident; and
- assist the RSO in maintaining records of sources and radioactive waste.

#### Radiological safety officer

A candidate with the following qualification is eligible to appear in the RSO Certification Examination conducted by the AERB.

#### Qualifications for RSO Level-II

- A post graduate degree/diploma in Nuclear Medicine recognized by the Medical Council of India or National Board of Examination, Ministry of Health and Family Welfare; Or
- A degree/post graduate diploma/post graduate degree in Nuclear Medicine Technology from an institution or university.

#### Responsibilities

RSO shall

- advise and assist the licensee to organize a radiation protection program appropriate for the facility and ensure that the staff observe safe work practices;
- ensure safety, security, and containment of radioactive sources, carry out radiation and contamination monitoring of work areas, patient waiting areas, radioactive waste disposal sites and public areas, and maintain records;
- ensure that radiation monitoring instruments are kept in proper working condition and are calibrated at regular intervals;
- establish procedures for management of emergency situations and conduct periodic drills to ensure their effectiveness;
- report any unusual incident in writing to the licensee, with a copy endorsed to the competent authority and take remedial measures to mitigate consequences of the incident and to prevent recurrence;
- maintain records of the doses of workers, the inventory of sources received, used, and disposed of, any unusual incident, cause of such incident, and remedial measures taken;
- ensure segregation and monitoring of the waste prior to interim storage or final disposal;
- advise and assist the licensee in ensuring regulatory compliance for obtaining authorization from the competent authority for procurement, use, transport, or disposal of radioactive material;
- inform the competent authority of his/her leaving the institution;
- advise and assist the licensee in transport of radioactive material/radioactive waste in the public domain;

ensure urgent processing of personnel dosimeters in cases of suspected overexposure; and display advisory notices in the NM departments to avoid unintentional exposures to pregnant women/lactating mothers.

## CONCLUSION

PET-CT facilities involve somewhat different design requirements than conventional NM facilities and are more likely to require additional shielding. By providing good handling facilities and following good work practices, radiation dose to the staff, public, and environment can be maintained well below the acceptable limit.

